# Friedel–Crafts-type reaction of pyrene with diethyl 1-(isothiocyanato)alkylphosphonates. Efficient synthesis of highly fluorescent diethyl 1-(pyrene-1-carboxamido)alkylphosphonates and 1-(pyrene-1-carboxamido)methylphosphonic acid

**DOI:** 10.3762/bjoc.11.266

**Published:** 2015-12-04

**Authors:** Anna Wrona-Piotrowicz, Janusz Zakrzewski, Anna Gajda, Tadeusz Gajda, Anna Makal, Arnaud Brosseau, Rémi Métivier

**Affiliations:** 1 Department of Organic Chemistry, Faculty of Chemistry, University of Łódź, Tamka 12, 91-403 Łódź, Poland; 2 Institute of Organic Chemistry, Faculty of Chemistry, Technical University of Łódź, Stefana Żeromskiego116, 90-924 Łódź, Poland; 3 University of Warsaw, Biological and Chemical Research Center, Żwirki i Wigury 101, 02-089 Warszawa, Poland; 4 PPSM, ENS Cachan, CNRS, UniverSud, 61 av President Wilson, 94230 Cachan, France

**Keywords:** amide, fluorescence, isothiocyanate, phosphonate, pyrene, thioamide, X-ray structure

## Abstract

Friedel–Crafts-type reaction of pyrene with diethyl 1-(isothiocyanato)alkylphosphonates promoted by trifluoromethanosulfonic acid afforded diethyl 1-(pyrene-1-carbothioamido)alkylphosphonates in 83–94% yield. These compounds were transformed, in 87–94% yield, into the corresponding diethyl 1-(pyrene-1-carboxamido)alkylphosphonates by treatment with Oxone^®^. 1-(Pyrene-1-carboxamido)methylphosphonic acid was obtained in a 87% yield by treating the corresponding diethyl phosphonate with Me_3_Si-Br in methanol. All of the synthesized amidophosphonates were emissive in solution and in the solid state. The presence of a phosphonato group brought about an approximately two-fold increase in solution fluorescence quantum yield in comparison with that of a model *N*-alkyl pyrene-1-carboxamide. This effect was tentatively explained by stiffening of the amidophosphonate lateral chain which was caused by the interaction (intramolecular hydrogen bond) of phosphonate and amide groups. The synthesized phosphonic acid was soluble in a biological aqueous buffer (PBS, 0.01 M, pH 7.35) and was strongly emissive under these conditions (λ_em_ = 383, 400 nm, τ = 18.7 ns, Φ_F_ > 0.98). Solid-state emission of diethyl 1-(pyrene-1-carboxamido)methylphosphonate (λ_max_ = 485 nm; Φ_F_ = 0.25) was assigned to π–π aggregates, the presence of which was revealed by single-crystal X-ray diffraction analysis.

## Introduction

Friedel–Crafts-type reaction of arenes with isothiocyanates constitutes a useful method for the synthesis of aromatic secondary thioamides [1–6]. Our group [7] and others [8] have recently described an efficient modification of this method by using trifluoromethanesulfonic (triflic) acid as a promoter. Furthermore, we reported a simple procedure for the oxidative desulfurization of thioamides to amides via reaction with Oxone^®^. Now we want to apply this approach to the synthesis of *N*-thioacyl- and acyl derivatives of 1-aminoalkylphosphonates from arenes and 1-(isothiocyanato)alkylphosphonates. 1-Aminoalkylphosphonates and their derivatives are compounds of biological relevance which have attracted the interest of biologically-oriented chemists and biochemists [9–12]. Furthermore, the phosphonate group offers various possibilities of metal binding or anchoring to solid surfaces [13–20]. For our purposes we chose pyrene as a model arene because of its high reactivity in reaction with simple isothiocyanates [7], and because the expected products, 1-(pyrene-1-carbothioamido)alkylphosphonates, should be easily transformed into the corresponding fluorescent amides [7,21–28]. Herein we report the results of this study along with the photophysical properties of the synthesized 1-(pyrene-1-carboxamido)alkylphosphonates.

## Results and Discussion

### Syntheses

**Scheme 1:**
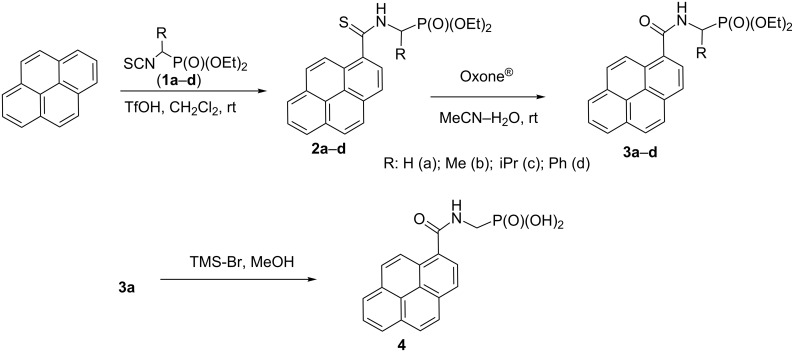
Synthesis of diethyl 1-(pyrene-1-carbothioamido)alkylphosphonates **2a–d**, diethyl 1-(pyrene-1-carboxamido)alkylphosphonates **3a–d** and (pyrene-1-carboxamido)methylphosphonic acid (**4**).

The reactions performed in this work are shown in Scheme 1.

We found that pyrene reacts with isothiocyanates **1a–d** in the presence of trifluoromethanesulfonic acid (TfOH) in dichloromethane at room temperature to afford 1-(pyrene-1-carbothioamido)alkylphosphonates **2a–d** in high (83–94%) isolated yields. These yields are comparable with those obtained using simple alkyl isothiocyanates [7], which means that the phosphonato group is perfectly compatible with the reaction conditions. The structures of **2a–d** were confirmed by spectroscopic and elemental analysis data and (for **3a**) by a single-crystal X-ray diffraction study (vide infra).

Thioamidophoshonates **2a–d** readily reacted with Oxone^®^ in acetonitrile–water at room temperature to afford the corresponding amidophosphonates **3a–d** in 87–94% isolated yield (Scheme 1). Therefore, the indirect route to these compounds described above proved very efficient. We did not attempt to prepare **3a–d** via direct Friedel–Crafts-type reaction of pyrene with (isocyanato)alkyl phosphonates because these compounds are difficult to synthesize and unstable [29]. Moreover, in our recent work we observed that reaction of pyrene with isothiocyanates proceeds more efficiently than an analogous reaction with isocyanates [7].

Additionally, compound **3a** was transformed, using a standard procedure [30], to the corresponding phosphonic acid **4** isolated in 87% yield.

### Photophysical properties of **3a**–**d** and **4**

**Figure 1:**
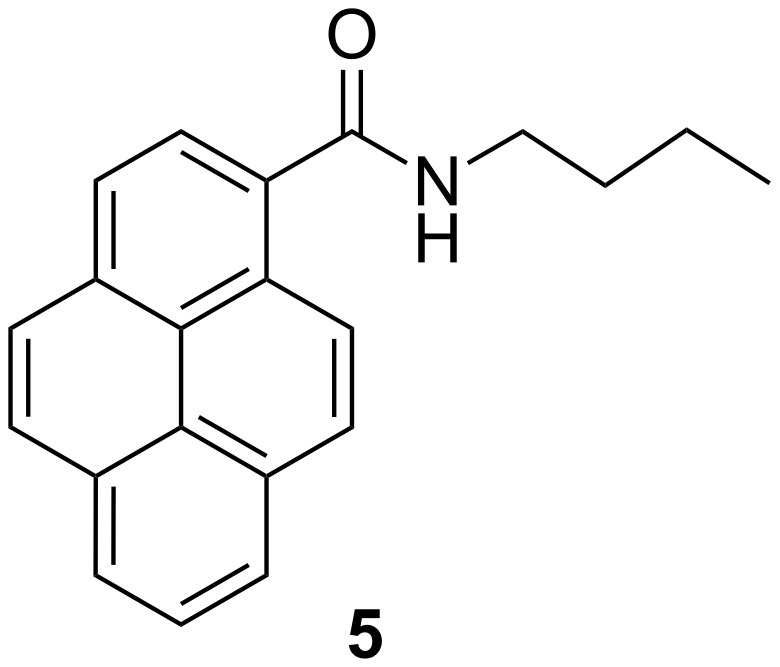
Structure of amide **5**.

As expected, thioamides **2a–d** were nonfluorescent (thioamide group is a well-known fluorescence quencher [31]). In contrast, the corresponding amides **3a–d** showed strong fluorescence emission in solution and in the solid state. We studied the photophysical properties of **3a–d** and **4** and, for comparison, those of *N*-alkylamide **5** (Figure 1).

The steady-state spectroscopic data for **3a–d**, **4** and **5**, along with fluorescence quantum yields in chloroform solutions, are presented in Table 1 (although it was recently reported that pyrene undergoes photodecomposition in this solvent [32], we found that this did not happen in the case of the compounds investigated here).

**Table 1:** Steady-state absorption and emission data for **3a**–**d**, **4**, **5** in CHCl_3_ solutions (*c* = 10^−6^ M).

Compound	Absorption λ_max_/nm (ε_max_/M^−1^ cm^−1^)	Emission^b^ λ_max_/nm	Φ_F_

**3a**	331 (27450), 345 (50910), 380 (960)	385, 405	0.68
**3b**	332 (38710), 345 (52480), 379 (4000)	385, 404	0.66
**3c**	331 (37850), 345 (51610), 379 (2750)	385, 404	0.63
**3d**	331 (37220), 345 (51400), 379 (4200)	387, 406	0.68
**4**^a^	327 (36350), 341 (50410), 376 (5100)	383, 400	>0.98
**5**	316 (16990), 329 (37400), 345 (52360), 377 (2030)	385, 403	0.34

^a^10^–6^ M solution in 0.01 M PBS (pH 7.35). ^b^λ_excit_ = 360 nm.

**Figure 2:**
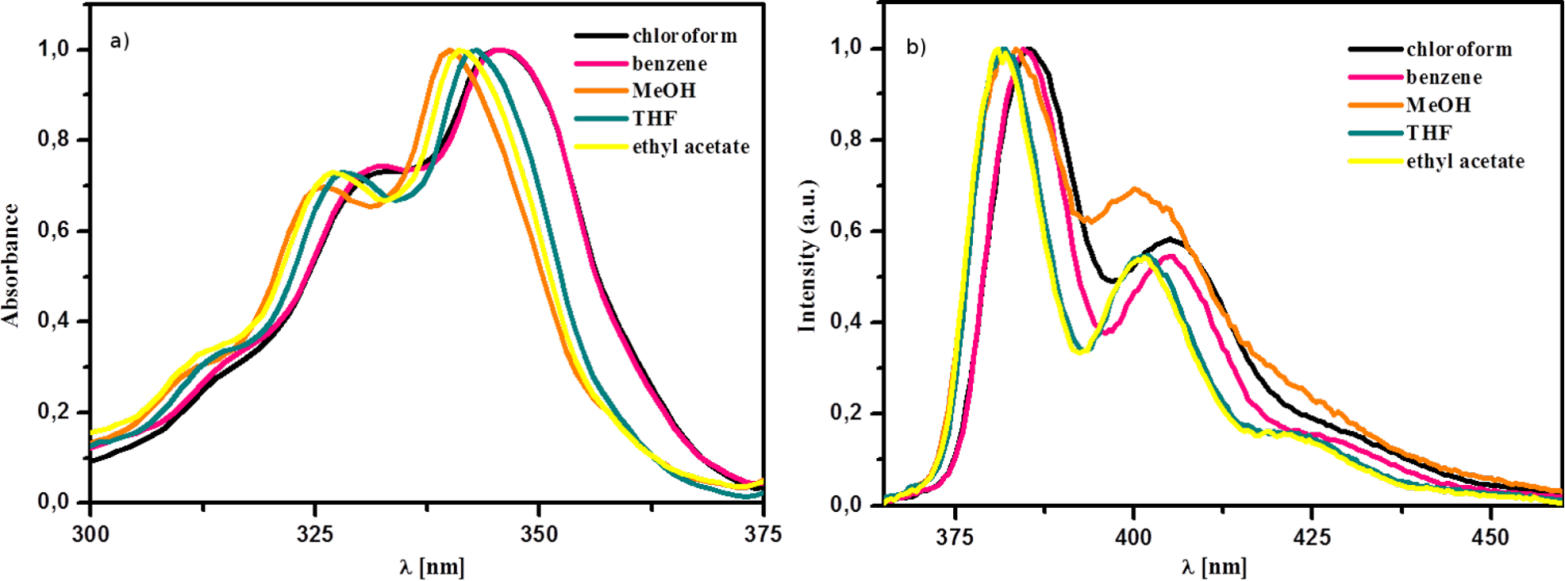
Normalized electronic absorption (a) and emission (b) spectra of **3a** in various solvents.

Electronic absorption and emission spectra of **3a** in various solvents are shown in Figure 2.

The introduction of a phosphonato group into the *N*-alkyl chain of the pyrene carboxamide fluorophore practically did not influence the electronic absorption and emission spectra. Structured emission bands were observed showing only a small effect of solvent polarity (Figure 1). However, the fluorescence quantum yields of **3a–d** and **4** were significantly higher than that of **5**.

**Figure 3:**
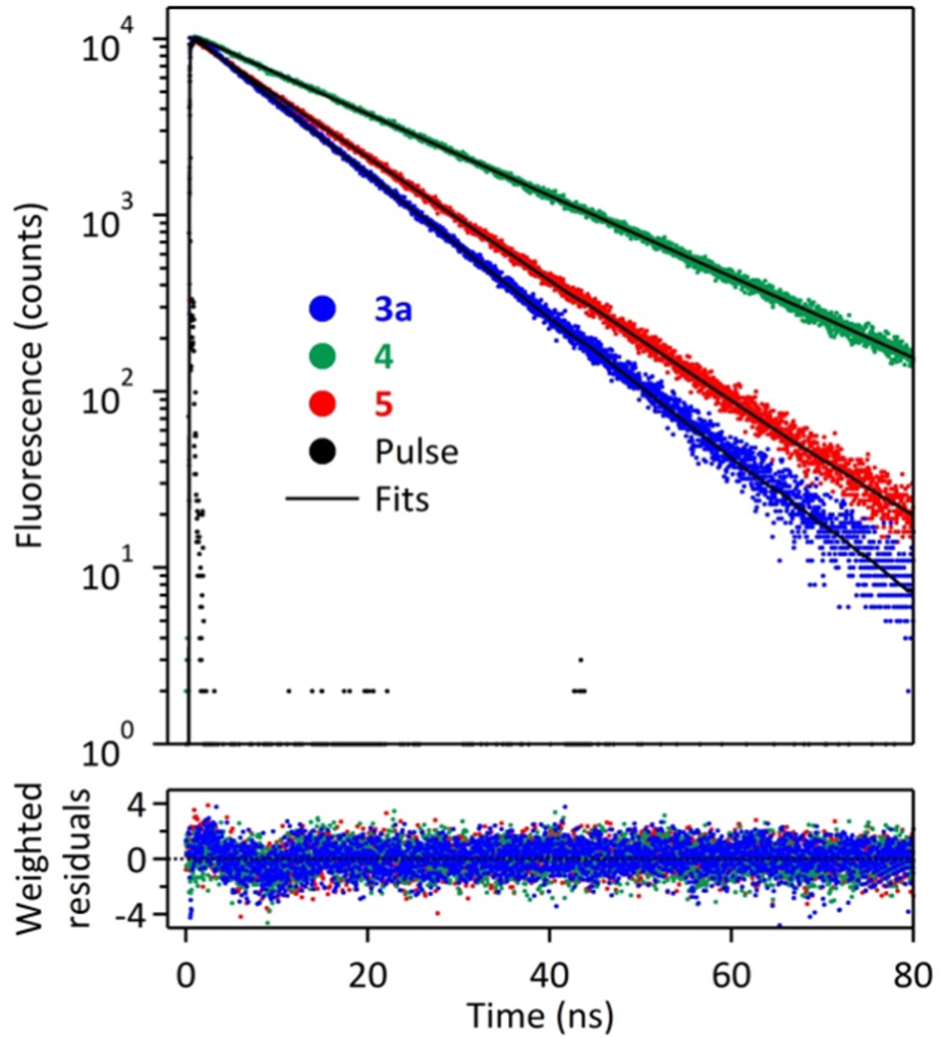
Fluorescence decay curves for **3a** and **5** in chloroform and for **4** in 0.01 M PBS (pH 7.35). λ_excit_ = 360 nm; λ_em_ = 385, 390 and 383 nm, respectively.

In order to explain this phenomenon we performed a time-resolved fluorescence study of **3a**, **4** and **5**. The fluorescence decay curves are shown in Figure 3, whereas the fluorescence lifetimes and decay rate constants are presented in Table 2. All of the compounds under study displayed monoexponential decays, thus indicating one emitting species.

**Table 2:** Fluorescence lifetimes and decay rate constants for **3a** and **5** in CHCl_3_ and for **4** in 0.01 M PBS (pH 7.35).

Compound	τ (ns); contribution	*k*_r_^a^ 10^7^ s^−1^	*k*_nr_^b^ 10^7^ s^−1^

**3a**	10.6 (1.00)	6.4	3.0
**4**	18.7 (1.00)	5.2	0.1
**5**	12.3 (1.00)	2.8	5.4

^a^*k*_r_ = Φ_F_ /τ; ^b^*k*_nr_ = (1−Φ_F_ )/τ.

Table 2 shows that **3a** displays a slightly shorter excited state lifetime (10.6 ns) than its alkyl analogue **5** (12.3 ns). The higher emission quantum yield of **3a** results from a ≈2.3 times higher value of *k*_r_ and a concomitant ca. 1.8 times lower value of *k*_nr_. Notably, compound **4** displays a *k*_r_ value that is comparable to that of **3a** (*k*_r_ = 5.3–6.4 × 10^7^ s^−1^ for the two species), but its non-radiative deactivation pathway is almost entirely suppressed. Therefore, it may be concluded that the phosphonato group exerts a significant influence on the excited state deactivation kinetics of **3a**.

**Figure 4:**
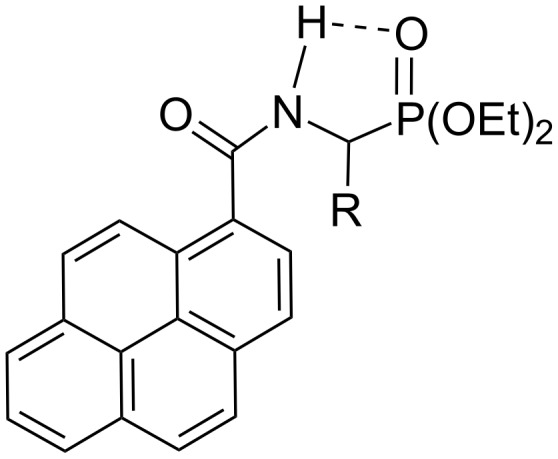
Intramolecular hydrogen bond in **3a–d**.

This influence may be attributed to the highly polar nature of the phosphonato group (electrostatic interaction between C=O and P=O dipoles may hamper rotations in the lateral chain responsible for non-radiative deactivation of the excited state). Another factor to be taken into consideration is the possibility of an intramolecular hydrogen bond between the N–H and P=O groups (Figure 4), which also leads to stiffening of the lateral chain and to blocking the non-radiative deactivation channel.

The presence of such a bond was verified via variable concentration ^1^H NMR spectroscopy [33], which showed only small variation (≈0.2 ppm) in the chemical shift of the N–H resonance of **3a** across a concentration range of 0.1–0.005 M in CDCl_3_ (see Supporting Information File 1).

Compound **4** was found to be insoluble in chloroform and in other weakly polar and nonpolar organic solvents but soluble in a biological buffer (0.01 M PBS, pH 7.35). Obviously, under these conditions it exists under an ionized form (anion or dianion). As was mentioned earlier, we observed efficient (Φ_F_ > 0.98) and relatively long-lived emission from **4** in the PBS buffer, which promises the possibility of its application in biological research.

**Figure 5:**
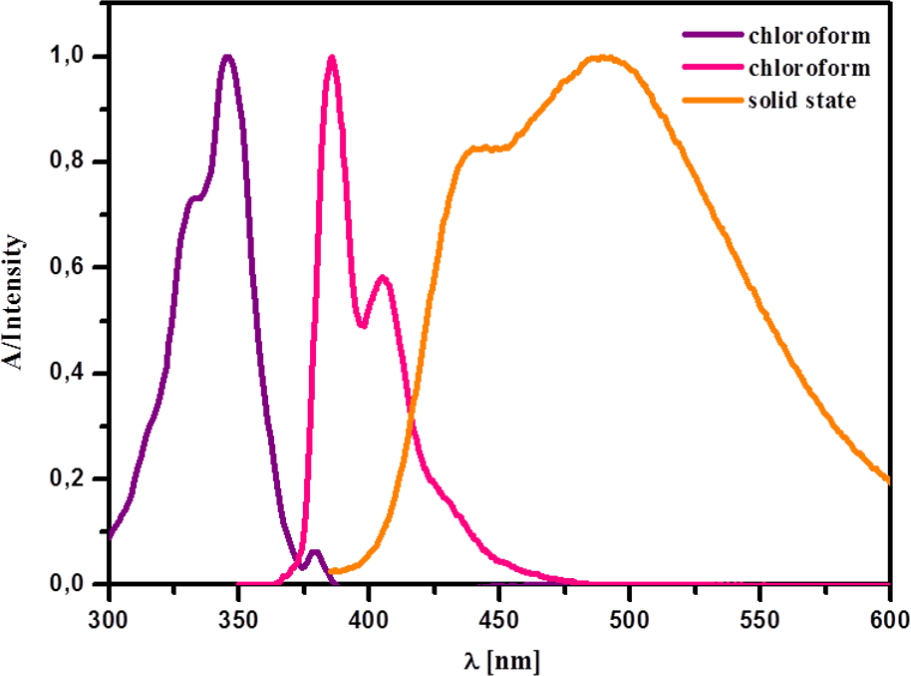
Normalized electronic absorption (violet) and emission (pink) spectrum of **3a** in CHCl_3_ (*c* = 10^−6^ M) and its solid-state emission spectrum (yellow). λ_excit_ = 360 nm.

Compound **3a** was also emissive in the solid state (λ_max_ = 485 nm; Φ_F_ = 0.25). The significant bathochromic shift (80 nm) and the broadness of the emission band in comparison with solution fluorescence (Figure 5) suggest the presence of emissive aggregates in the solid state. This was confirmed by an X-ray diffraction study (vide infra).

### Molecular structure, crystal packing and solid-state fluorescence of **3a**

**Figure 6:**
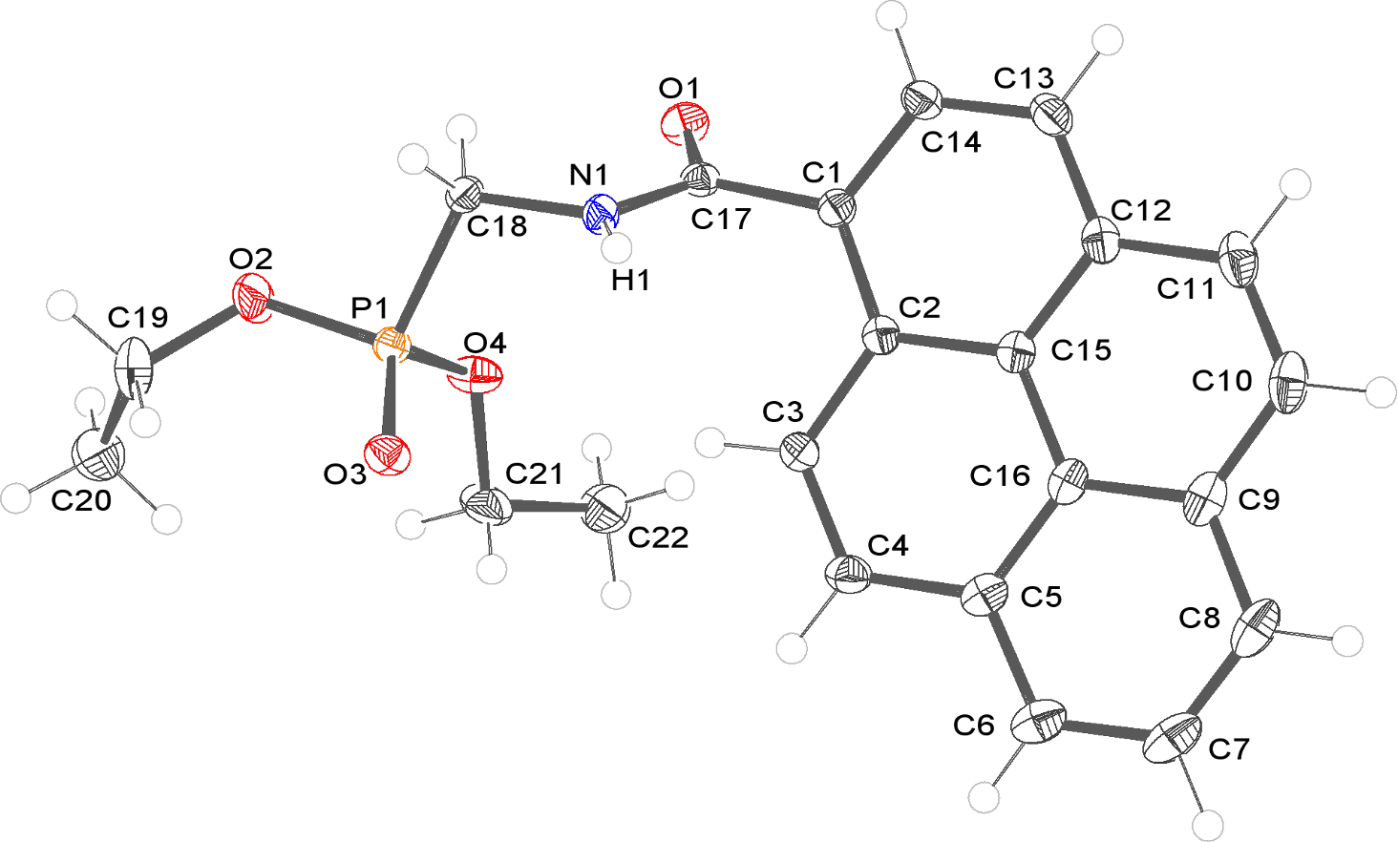
Molecular structure of **3a** (ORTEP representation). Displacement ellipsoids were drawn at a 50% probability level. Hydrogen atom labels are analogous to those of bonded non-H atoms and were omitted for clarity, apart from the H1 atom which was involved in intermolecular hydrogen bond formation.

Compound **3a** crystallized from layered dichloromethane–hexane in the monoclinic space group *P*2_1_/*c*, with a single molecule occupying an asymmetric unit in general position. Its molecular structure is shown in Figure 6.

The pyrenyl moiety in **3a** is slightly bent, the angle between the planes of rings C1–C2–C15–C14–C13–C12 and C5–C6–C7–C8–C9–C16 is 4.23(13)°. The amide group is twisted by 57.1(2) degrees from the plane of the C1–C2–C15–C14–C13–C12 ring. Together with the C1–C17 bond length exceeding 1.5 Å, this suggests that there is only very weak electronic conjugation between these units. The phosphonato group is also almost perpendicular to the plane of the amide group; the relevant C17–N1–C18–P1 torsion angle is 102.39(13)°. The most important geometrical parameters and intermolecular contacts for the structure are summarized in Table 3 and Table 4, respectively.

**Table 3:** Selected geometrical parameters for **3a**.

Bond lengths	Å

P1–O2	1.5761(11)
P1–O3	1.4751(10)
P1–O4	1.5694(11)
P1–C18	1.8000(14)
C1–C17	1.5053(18)
O1–C17	1.2315(16)
N1–C18	1.4500(17)
N1–C17	1.3463(17)

Torsion angles	°

C12–C15–C16–C5	−179.43(12)
C12–C15–C16–C9	−0.42(19)
C2–C1–C17–O1	−123.81(15)
C2–C1–C17–N1	58.89(17)
C17–N1–C18–P1	102.39(13)

**Table 4:** Selected hydrogen bonds in the crystal structure of **3a**.

D	H	A	D–H [Å]	D–H [Å]	D–H [Å]	D–H … A [ ° ]	Symmetry operation for A atom

N1	H1	O3	0.871(14)	1.941(14)	2.7991(15)	168.0(16)	1−x, −y, 1−z
C20	H20C	O1	0.981	2.683	3.369(2)	127.3	1−x, −½+y, ½−z
C21	H21A	O1	0.990	2.6145	3.585(2)	166.6	1−x, −½+y, ½−z
C22	H22C	O3	0.980	2.708	3.664(2)	165.2	1−x, ½+y, ½−z

The molecules **3a** form dimers in the crystal related by the crystallographic center of symmetry, bound by intermolecular N1–H1···O3 hydrogen bonds and stabilized by C19–H19B···π interactions. Similar NH···O=P hydrogen-bonded dimers were already observed in the crystals of some aminophosphonates [34].

**Figure 7:**
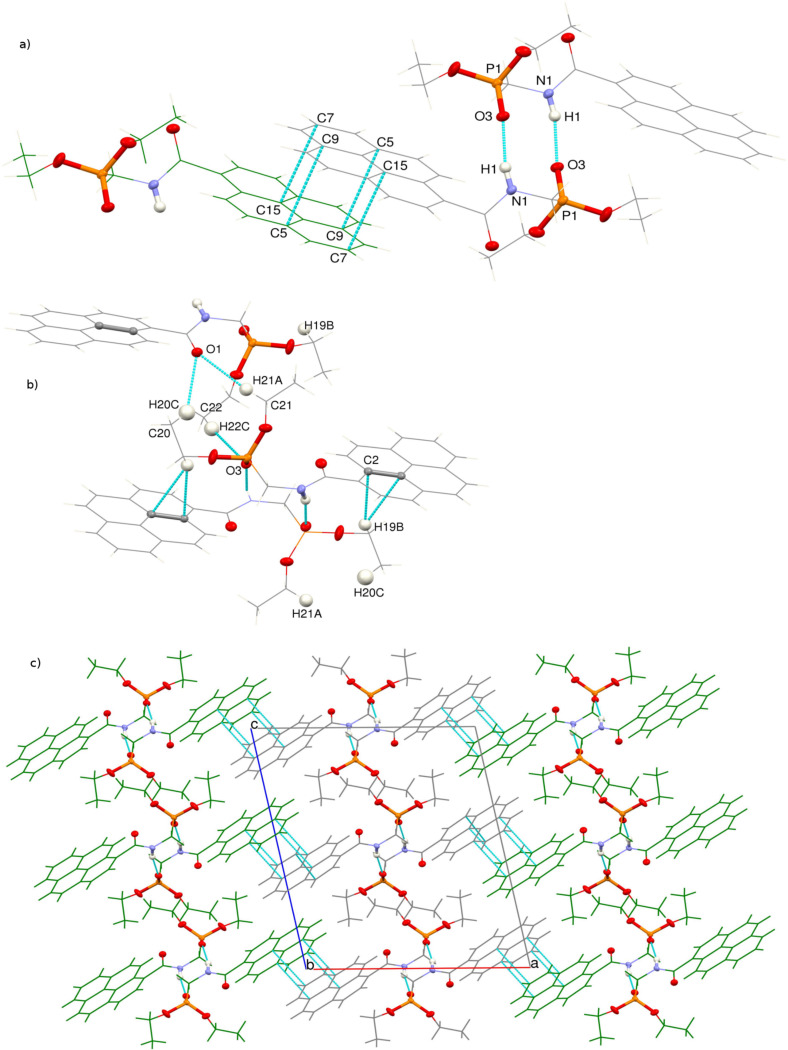
(a) Dimers of π-stacked and hydrogen-bonded molecules of **3a** represented in single figures; (b) network of weak C–H^…^O and C–H^…^π interactions stabilizing H-bonded dimer strings and (c) crystal packing along the crystallographic b direction. Ellipsoids are drawn at 50% probability level for all non-C and non-H atoms. The remaining atoms are represented as wires in grey or green in order to differentiate between adjacent π^…^π interacting chains of H-bonded atoms. The shortest intermolecular contacts are represented in cyan.

The dimers form strings along the crystallographic [001] direction, bound by relatively short (H···O distances of less than 2.75 Å) and intermolecular hydrogen C–H···O bonds (Table 4). On the other hand, the pyrenyl moieties from the adjacent strings along [100] take part in π···π interactions, also forming molecular dimers (Figure 7). The shortest C···C distances are C7–C15 and C5–C9 (3.367(2) Å, and 3.410(2) Å, respectively).

The presence of π–π-bonded aggregates in the crystal structure of **3a** is in line with the observed features of solid-state emission of this compound (vide suppra).

## Conclusion

We demonstrated the feasibility of the Friedel–Crafts reaction with 1-(isothiocyanato)alkylphosphonates by using pyrene as an arene. The pyrenyl thioamidoalkylphosphonates formed in this reaction can be readily transformed into the corresponding fluorescent amidoalkylphosphonates. It is worthy to note that this class of compounds offers numerous possibilities of chemical transformations [35–36]. The pyrenyl amidoalkylphosphonates emit fluorescence with quantum yields ca. 2 times higher than simple *N*-substituted pyrene-1-carboxamides. Solid-state emission assigned to the aggregates was also observed. Finally, a pyrenecarboxamide phosphonic acid was synthesized, showing very efficient emission in a biological buffer and promising possible biological applications.

## Supporting Information

File 1 Experimental procedures, characterization of new compounds, and details of the photophysical study.

File 2 CIF file of compound **3a**.
