# Supplementary material for: Friedel–Crafts-type reaction of pyrene with diethyl 1-(isothiocyanato)alkylphosphonates. Efficient synthesis of highly fluorescent diethyl 1-(pyrene-1-carboxamido)alkylphosphonates and 1-(pyrene-1-carboxamido)methylphosphonic acid
Source: Beilstein J Org Chem. 2015 Dec 4;11:2451–8. doi: 10.3762/bjoc.11.266 (PMC4685883; doi:10.3762/bjoc.11.266)
Supplement: File 1 — Experimental procedures, characterization of new compounds, and details of the photophysical study. [file Beilstein_J_Org_Chem-11-2451-s001.pdf]

Supporting Information

for

Friedel–Crafts-type reaction of pyrene with diethyl 1-(isothiocyanato)alkylphosphonates. Efficient synthesis of highly fluorescent diethyl 1-(pyrene-1-carboxamido)alkylphosphonates and 1-(pyrene-1-carboxamido)methylphosphonic acid

Anna Wrona-Piotrowicz[1], Janusz Zakrzewski[1*], Anna Gajda[2], Tadeusz Gajda[2], Anna Makal[3], Arnaud Brosseau[4], Rémi Métivier[4]

Address: [1]Department of Organic Chemistry, Faculty of Chemistry, University of Łódź, Tamka 12, 91-403 Łódź, Poland, [2]Institute of Organic Chemistry, Faculty of Chemistry, Technical University of Łódź, Stefana Żeromskiego116, 90-924 Łódź, Poland, [3]University of Warsaw, Biological and Chemical Research Center, Żwirki i Wigury 101, 02-089 Warszawa, Poland and [4]PPSM, ENS Cachan, CNRS, UniverSud, 61 av President Wilson, 94230 Cachan, France

Email: Janusz Zakrzewski – janzak@uni.lodz.pl

*Corresponding author

Experimental procedures, characterization of new compounds, and details of the photophysical study

1. Experimental section …………………………………………………………………... s2

1.1. General ……………………………………………………………...….... s2

1.2. Syntheses of diethyl 1-(pyrene-1-carbothioamido)alkylphosphonates **2a–d** …... s3

1.3. Syntheses of diethyl 1-(pyrene-1-carboxamido)alkylphosphonates **3a–d** ……... s4

1.4. Synthesis of 1-(pyrene-1-carboxamido)methylphosphonic acid **4** ……………... s5

2. X-ray diffraction analysis of diethyl 1-(pyrene-1-carboxamido)methylphosphonate **3a** s6

3. Fig.S1. Variable concentration ^{1}H NMR spectra of **3a** in $CDCl_3$ ...……………………..….. s8

4. Photophysical study ...…………………………………………………………………….. s8

5. References ...……………………………………………………………………………..... s9

1.Experimental section

1.1. General

Solvents were purified before use by reported methods. Diethyl 1-(isothiocyanato)alkylphosphonates **1a–d** were synthesized according to an earlier published procedure [1,2]. All reagents were purchased from Sigma-Aldrich and used without further purification. Column chromatography was carried out on silica gel 60 (0.040–0.063 mm, 230-400 mesh, Fluka). ^{1}H and ^{13}C NMR spectra were recorded in $CDCl_3$ on a Bruker ARX 600 MHz Spectrometer (600 MHz for ^{1}H) at room temperature. Chemical shifts were referenced relative to solvent signals (^{1}H and ^{13}C spectra) and to ext. 85% H_3PO_4 (^{31}P spectra). IR spectra were run on a FTIR Nexus spectrometer in KBr pellets. Elemental analyses were performed in the Laboratory of Microanalysis at The Centre of Molecular and Macromolecular Studies in Łódź**,** Poland.

1.2 Syntheses of diethyl 1-(pyrene-1-carbothioamido)alkylphosphonates 2a–d

These compounds were prepared in a similar manner as described before [3].

Diethyl 1-(isothiocyanato)alkylphosphonate (1.5 mmol) and TfOH (348 μL, 4 mmol) were added at room temperature to a solution of pyrene (202 mg, 1 mmol) in CH_2Cl_2 (10 mL). After stirring for 2 h the reaction mixture was poured onto ice-water (50 mL) and extracted several times with CH_2Cl_2. The combined extracts were dried (Na_2SO_4) and evaporated to dryness. Flash chromatography (silica gel/ CH_2Cl_2) afforded pure products.

2a. Yellow powder (387 mg, 94%), m.p. 201 - 202 °C; ^{1}H NMR (600 MHz, $CDCl_3$): δ 8.36 (s, 1H), 8.33 (d, J = 9.6 Hz, 1H), 8.22 (d, J = 7.8 Hz, 1H), 8.20 (d, J = 7.2 Hz, 1H), 8.15 (d, J = 7.8 Hz, 1H), 8.11 (d, J = 7.8 Hz, 2H), 8.06 (d, J = 7.8 Hz, 1H), 8.05 (d, J = 9.0 Hz, 1H), 8.04 (t, J = 7.8 Hz, 1H), 4.52 (dd, J_1 = 16.8 Hz, J_2 = 7.2 Hz, 2H), 4.04 – 4.09 (m, 4H), 1.27 – 1.29 (m, 6H); ^{13}C NMR (150 MHz, $CDCl_3$),: δ 16.36 (d, J = 6 Hz), 41.05 (d, J = 153 Hz), 62.90 (d, J = 7.5 Hz), 123.80, 124.52, 124.57, 124.70, 125.01, 125.59, 125.90, 126.40, 126.50, 127.17, 128.45, 128.59, 130.73, 131.32, 132.04, 138.10, 201.84 (d, J = 7.5 Hz); ^{31}P NMR (243 MHz, $CDCl_3$): δ 20.47; IR (KBr): 3163, 3012, 2983, 2090, 1543, 1240, 1024, 842 cm^{-1}; Anal. calcd for $C_{22}H_{22}NO_3PS$: C, 64.22; H, 5.39; N, 3.40; S, 7.79; Found: C, 64.17; H, 5.45; N, 3.46; S, 7.68;

2b. Yellow powder (353 mg, 83%), m.p. 209 - 210 °C; ^{1}H NMR (600 MHz, $CDCl_3$): δ 9.26 (d, J = 8.4 Hz, 1H), 8.28 (d, J = 9.6 Hz, 1H), 8.18 (d, J = 7.8 Hz, 1H), 8.17 (d, J = 7.8 Hz, 1H), 8.10 (d, J = 8.4 Hz, 1H), 8.07 (d, J = 7.2 Hz, 1H), 8.05 (d, J = 7.8 Hz, 1H), 7.99 – 8.02 (m, 3H), 5.57 – 5.62 (m, 1H), 3.87 – 3.91 (m, 2H), 3.46 – 3.50 (m, 2H), 1.49 (dd, J_1 = 16.8 Hz, J_2 = 7.2 Hz, 3H), 1.17 (t, J = 7.2 Hz, 3H), 0.90 (t, J = 7.2 Hz, 3H); ^{13}C NMR (150 MHz, $CDCl_3$),: δ 14.25, 15.93 (d, J = 6 Hz), 16.29 (d, J = 6 Hz), 47.51 (d, J = 155 Hz), 62.45 (d, J = 6 Hz), 62.67 (d, J = 7.5 Hz), 123.97, 124.31, 124.48, 124.54, 124.82, 125.33, 125.66, 126.25, 126.31, 127.07, 128.12, 128.20, 130.68, 131.21, 131.64, 138.46, 200.74 (d, J = 6 Hz); ^{31}P NMR (243 MHz, $CDCl_3$): δ 23.03; IR (KBr): 3165, 3009, 2980, 2097, 1537, 1238, 1030, 841 cm^{-1}; Anal. calcd for $C_{23}H_{24}NO_3PS$: C, 64.93; H, 5.69; N, 3.29; S, 7.54; Found: C, 64.90; H, 5.77; N, 3.32; S, 7.46;

2c. Yellow powder (381 mg, 84%), m.p. 184 - 185 °C; ^{1}H NMR (600 MHz, $CDCl_3$): δ 8.43 (d, J = 10.2 Hz, 1H), 8.39 (d, J = 9.6 Hz, 1H), 8.19 (t, J = 7.8 Hz, 2H), 8.13 (d, J = 7.8 Hz, 1H), 8.09 (d, J = 9.0 Hz, 1H), 8.08 (d, J = 9.0 Hz, 1H), 8.00 – 8.04 (m, 2H), 7.99 (d, J = 7.8 Hz, 1H), 5.64 – 5.70 (m, 1H), 4.06 – 4.12 (m, 2H), 3.94 – 3.97 (m, 2H), 2.40 – 2.46 (m, 1H), 1.28 (t, J = 7.2 Hz, 3H), 1.22 (t, J = 7.2 Hz, 3H), 1.21 (d, J = 7.8 Hz, 3H), 1.13 (d, J = 7.2 Hz, 3H); ^{13}C NMR (150 MHz, $CDCl_3$),: δ 16.24 (d, J = 6 Hz), 16.41 (d, J = 6 Hz), 18.71 (d, J = 6 Hz), 20.74 (d, J = 10.5 Hz), 29.96 (d, J = 3 Hz), 56.60 (d, J = 148 Hz), 62.25 (d, J = 7.5 Hz), 62.71 (d, J = 4.5 Hz), 124.02, 124.43, 124.47, 124.52, 124.67, 125.42, 125.72, 126.30, 126.41, 127.06, 128.24, 128.31, 130.74, 131.25, 131.74, 138.70, 202.33 (d, J = 7.5 Hz); ^{31}P NMR (243 MHz, $CDCl_3$): δ 21.93; IR (KBr): 3161, 2980, 2904, 2074, 1541, 1378, 1228, 1023, 844 cm^{-1}; Anal. calcd for $C_{25}H_{28}NO_3PS$: C, 66.21; H, 6.22; N, 3.09; S, 7.07; Found: C, 66.24; H, 6.25; N, 3.00; S, 7.12;

2d. Yellow powder (444 mg, 91%), m.p. 227 - 228 °C; ^{1}H NMR (600 MHz, $CDCl_3$): δ 10.08 (s, 1H), 8.18 (d, J = 7.2 Hz, 1H), 8.14 (d, J = 7.8 Hz, 1H), 8.11 (d, J = 9.6 Hz, 1H), 8.07 (d, J = 9.0 Hz, 1H), 8.06 (d, J = 7.8 Hz, 1H), 8.03 (t, J = 7.8 Hz, 1H), 8.02 (d, J = 7.8 Hz, 1H), 7.96 (d, J = 9.0 Hz, 1H), 7.93 (d, J = 7.8 Hz, 1H), 7.58 – 7.60 (m, 2H), 7.38 – 7.40 (m, 3H), 6.65 (dd, J_1 = 20.4 Hz, J_2 = 9.6 Hz, 1H), 3.72 – 3.75 (m, 2H), 3.05 – 3.17 (m, 2H), 1.27 (t, J = 7.2 Hz, 3H), 1.15 (t, J = 7.2 Hz, 3H); ^{13}C NMR (150 MHz, $CDCl_3$),: δ 15.58 (d, J = 7.5 Hz), 16.27 (d, J = 4.5 Hz), 56.40 (d, J = 154,5 Hz), 62.67 (d, J = 7.5 Hz), 124.12, 124.25, 124.51 (d, J = 6 Hz), 124.71, 125.33, 125.63, 126.25, 126.52, 127.07, 127.40 (d, J = 6 Hz), 128.12 (d, J = 3 Hz), 128.36 (d, J = 1.5 Hz), 128.74, 128.86, 128.95 (d, J = 6 Hz), 130.72, 131.23, 131.62, 133.78, 138.37, 201.34 (d, J = 12 Hz); ^{31}P NMR (243 MHz, $CDCl_3$): δ 19.03; IR

(KBr): 3161, 2983, 2926, 2063, 1540, 1373, 1237, 1025, 847 cm^{-1}; Anal. calcd for $C_{28}H_{26}NO_3PS$: C, 68.98; H, 5.38; N, 2.87; S, 6.58; Found: C, 68.87; H, 5.41; N, 2.99; S, 6.48;

1.3.Syntheses of diethyl 1-(pyrene-1-carboxamido)alkylphosphonates 3a–d

These compounds were prepared in a similar manner as described before [3].

Oxone® (1.5 mmol in 10 mL of water) was added to the thioamides **2a–d** (1 mmol) dissolved in a mixture of acetonitrile (30 mL) and water (20 mL). The resulting solution was stirred at room temperature overnight, poured into water (100 mL) and extracted several times with CH_2Cl_2. The organic phase was dried over anhydrous Na_2SO_4 and concentrated. The products were purified by silica gel column chromatography (CH_2Cl_2/MeOH 100 : 0 /99 : 1).

3a. White powder (356 mg, 90%), m.p. 108 - 109 °C; ^{1}H NMR (600 MHz, $CDCl_3$): δ 8.51 (d, J = 9.6 Hz, 1H), 8.14 (t, J = 7.2 Hz, 2H), 8.04 (d, J = 9.0 Hz, 1H), 8.03 (d, J = 7.2 Hz, 1H), 8.02 (d, J = 7.2 Hz, 1H), 8.01 (d, J = 7.8 Hz, 1H), 7.98 (t, J = 7.2 Hz, 1H), 7.92 (d, J = 9.0 Hz, 1H), 7.12 (s, 1H), 4.06 – 4.11 (m, 4H), 4.01 (dd, J_1 = 12 Hz, J_2 = 6 Hz, 2H), 1.27 (t, J = 7.2 Hz, 6H); ^{13}C NMR (150 MHz, $CDCl_3$),: δ 16.28 (d, J = 6 Hz), 35.40 (d, J = 154.5 Hz), 62.55 (d, J = 7.5 Hz), 124.08, 124.18, 124.51, 124.62, 125.60, 125.73, 126.18, 126.89, 128.52, 128.55, 128.58, 130.01, 130.50, 130.97, 132.53, 169.62 (d, J = 4.5 Hz); ^{31}P NMR (243 MHz, $CDCl_3$): δ 22.59; IR (KBr): 3233, 3050, 2980, 1908, 1663, 1550, 1243, 1209, 1027, 846 cm^{-1}; Anal. calcd for $C_{22}H_{22}NO_4P$: C, 66.83; H, 5.61; N, 3.54; Found: C, 66.75; H, 5.66; N, 3.61;

3b. White powder (377 mg, 92%), m.p. 140.5 - 141 °C; ^{1}H NMR (600 MHz, DMSO-d_6): δ 9.01 (d, J = 9.0 Hz, 1H), 8.51 (d, J = 9.0 Hz, 1H), 8.35 (t, J = 7.8 Hz, 3H), 8.26 (d, J = 9.0 Hz, 2H), 8.22 (d, J = 9.0 Hz, 1H), 8.12 (t, J = 7.8 Hz, 1H), 8.09 (d, J = 7.8 Hz, 1H), 4.69 – 4.75 (m, 1H), 4.13 – 4.18 (m, 4H), 1.44 (dd, J_1 = 16.8 Hz, J_2 = 7.8 Hz, 3H), 1.32 (t, J = 7.2 Hz, 3H), 1.31 (t, J = 7.2 Hz, 3H); ^{13}C NMR (150 MHz, DMSO-d_6),: δ 14.85, 16.28 (d, J = 6 Hz), 16.36 (d, J = 6 Hz), 41.60 (d, J = 156 Hz), 61.74 (d, J = 6 Hz), 61.87 (d, J = 7.5 Hz), 123.56, 123.66, 124.23, 124.54, 125.15, 125.52, 125.71, 126.47, 127.10, 127.75, 127.91, 128.21, 130.13, 130.63, 131.52, 131.53, 168.12 (d, J = 6 Hz); ^{31}P NMR (243 MHz, DMSO-d_6): δ 24.08; IR (KBr): 3239, 3041, 2983, 2083, 1660, 1533, 1229, 1050, 1018,973, 851 cm^{-1}; Anal. calcd for $C_{23}H_{24}NO_4P$: C, 67.47; H, 5.91; N, 3.42; Found: C, 67.40; H, 6.01; N, 3.45;

3c. White powder (411 mg, 94%), m.p. 147 - 148 °C; ^{1}H NMR (600 MHz, DMSO-d_6): δ 8.92 (d, J = 10.2 Hz, 1H), 8.45 (d, J = 9.0 Hz, 1H), 8.35 (t, J = 7.2 Hz, 3H), 8.26 (d, J = 9.0 Hz, 2H), 8.23 (d, J = 9.0 Hz, 1H), 8.12 (t, J = 7.8 Hz, 1H), 8.07 (d, J = 7.8 Hz, 1H), 4.51 – 4.57

(m, 1H), 4.12 – 4.17 (m, 4H), 2.21 – 2.26 (m, 1H), 1.33 (t, *J* = 7.2 Hz, 3H), 1.29 (t, *J* = 7.2 Hz, 3H), 1.15 (d, *J* = 6.6 Hz, 3H), 1.10 (d, *J* = 6.6 Hz, 3H); ^{13}C NMR (150 MHz, DMSO-d_6),: δ 16.24 (d, *J* = 6 Hz), 16.34 (d, *J* = 6 Hz), 19.05 (d, *J* = 7.5 Hz), 20.45 (d, *J* = 7.5 Hz), 28.45 (d, *J* = 3 Hz), 51.01 (d, *J* = 151.5 Hz), 61.52 (d, *J* = 6 Hz), 61.65 (d, *J* = 7.5 Hz), 123.58, 123.65, 124.23, 124.50, 125.11, 125.50, 125.70, 126.47, 127.11, 127.70, 127.90, 128.16, 130.14, 130.63, 131.47, 131.79, 169.00 (d, *J* = 6 Hz); ^{31}P NMR (243 MHz, DMSO-d_6): δ 24.54; IR (KBr): 3227, 3040, 2973, 2926, 2907, 1655, 1529, 1298, 1233, 1028, 846 cm^{-1}; Anal. calcd for $C_{25}H_{28}NO_4P$: C, 68.64; H, 6.45; N, 3.20; Found: C, 68.63; H, 6.48; N, 3.11;

3d. White powder (410 mg, 87%), m.p. 186 - 187 °C; ^{1}H NMR (600 MHz, $CDCl_3$): δ 8.50 (d, *J* = 9.0 Hz, 1H), 8.19 (t, *J* = 7.2 Hz, 2H), 8.06 – 8.10 (m, 4H), 8.02 (t, *J* = 7.8 Hz, 2H), 7.76 – 7.77 (m, 1H), 7.63 (d, *J* = 7.8 Hz, 2H), 7.41 (t, *J* = 7.8 Hz, 2H), 7.36 (d, *J* = 7.8 Hz, 1H), 5.94 (dd, J_1 = 21.0 Hz, J_2 = 8.4 Hz, 1H), 4.00 – 4.06 (m, 2H), 3.76 – 3.82 (m, 1H), 3.62 – 3.66 (m, 1H), 1.25 (t, *J* = 7.2 Hz, 3H), 1.02 (t, *J* = 7.2 Hz, 3H); ^{13}C NMR (150 MHz, $CDCl_3$),: δ 16.03 (d, *J* = 4.5 Hz), 16.35 (d, *J* = 4.5 Hz), 50.81 (d, *J* = 153 Hz), 62.84 (d, *J* = 7.5 Hz), 63.34 (d, *J* = 6 Hz), 124.15, 124.34 (d, *J* = 6 Hz), 124.66, 124.78, 125.68, 125.79, 126.27, 127.02, 128.17 (d, *J* = 1.5 Hz), 128.32 (d, *J* = 6 Hz), 128.60, 128.69 (d, *J* = 1.5 Hz), 128.76, 130.28, 130.64, 131.10, 132.64, 135.19, 169.12 (d, *J* = 7.5 Hz); ^{31}P NMR (243 MHz, $CDCl_3$): δ 21.30; IR (KBr): 3251, 3037, 2930, 1652, 1529, 1244, 1026, 849 cm^{-1}; Anal. calcd for $C_{28}H_{26}NO_4P$: C, 71.33; H, 5.56; N, 2.97; Found: C, 71.35; H, 5.59; N, 2.91;

1.4. Synthesis of 1-(pyrene-1-carboxamido)methylphosphonic acid 4

Compound **3a** (395 mg, 1 mmol) was dissolved in anhydrous dichloromethane (5 mL) and Me_3SiBr (3.5 mmol) was added. The mixture was stirred overnight at room temperature. After this time the solvent was evaporated and the residue was dissolved in methanol. The mixture was allowed to stir at room temperature overnight. The white crude product was purified by silica gel column chromatography (CH_2Cl_2/MeOH 95 : 5).

4. White powder (295 mg, 87%), m.p. 257 – 258 °C; ^{1}H NMR (600 MHz, DMSO-d_6): δ 8.79 (t, *J* = 6 Hz, 1H), 8.56 (d, *J* = 9.6 Hz, 1H), 8.34 (d, *J* = 7.8 Hz, 2H), 8.32 (d, *J* = 7.8 Hz, 1H), 8.21 – 8.26 (m, 3H), 8.15 (d, *J* = 7.8 Hz, 1H), 8.11 (t, *J* = 7.8 Hz, 1H), 3.75 (dd, J_1 = 12 Hz, J_2 = 6 Hz, 2H); ^{13}C NMR (150 MHz, DMSO-d_6),: δ 37.10 (d, *J* = 151.5 Hz), 123.57, 123.69, 124.21, 124.90, 125.29, 125.48, 125.64, 126.44, 127.12, 127.83, 127.85, 128.16, 130.16, 130.63, 131.51, 131.73, 168.44 (d, *J* = 4.5 Hz); ^{31}P NMR (243 MHz, DMSO-d_6): δ 18.03; IR

(KBr): 3423, 3293, 3045, 1647, 1549, 1227, 1013, 854 cm^{-1}; Anal. calcd for $C_{18}H_{14}NO_4P$: C, 63.72; H, 4.16; N, 4.13; Found: C, 63.69; H, 4.25; N, 4.20;

2. X-ray diffraction analysis of diethyl 1-(pyrene-1-carboxamido)methylphosphonate 3a

Crystals suitable for X-ray analysis were obtained by slow diffusion of pentane into a solution of this compound in dichloromethane at room temperature. The crystals were clear colorless plates.

The data for were collected on Agilent Supernova 4 circle diffractometer system equipped with molybdenum microsource and Eos CCD detector. The data were collected using MoKα radiation with CrysAlis171 software and integrated with the CrysAlisPRO software. Data were corrected for absorption effects using the numerical methods (SCALE3 ABSPACK).

The structure was solved by direct methods using SXELXS and refined by the full-matrix least squares procedure with SHELXL within the OLEX2 graphical interface. Figures were produced with Ortep3v2 and Mercury_3.3 software.

All H atoms were visible in the residual density map, but were added geometrically and refined in riding approximation. Positions of the H atoms involved in hydrogen bonds were refined with restraint applied to the N–H distance as suggested by SHELXL for 100 K.

The quantitative descriptors of the data processing and structure refinement for all compounds are presented in Table S1.

Table S1:

**Identification code**	**3a**
Empirical formula	$C_{22}H_{22}NO_4P$
Formula weight	395.37
Temperature/K	100.00(10)
Crystal system	monoclinic
Space group	$P2_1/c$
a/Å	14.1565(2)
b/Å	8.31073(11)
c/Å	16.8189(3)
α/°	90
β/°	101.4970(15)

γ/°	90
Volume/Å^3	1939.06(5)
Z	4
ρ_{calc}mg/mm^3	1.354
μ/mm^{-1}	0.17
F(000)	832
Crystal size/mm^3	0.33×0.19×0.03
Radiation Å	MoKα (λ = 0.71073)
2Θ range for data collection	2.47° to 65.12°
Index ranges	-21 ≤ h ≤ 21, -12 ≤ k ≤ 12, -23 ≤ l ≤ 25
Reflections collected	30281
Independent reflections	6669
Rint	0.045
Rsigma	0.042
Data/restraints /parameters	6669/1/258
Goodness-of-fit on F^2	1.043
Final R indexes [I>=2σ (I)]	R1 = 0.0468, wR2 = 0.1130
Final R indexes [all data]	R1 = 0.0683, wR2 = 0.1249
Largest diff. peak/hole / e Å^{-3}	0.523/-0.445

3. Figure S1: Variable concentration [1]NMR spectra of **3a** in $CDCl_3$

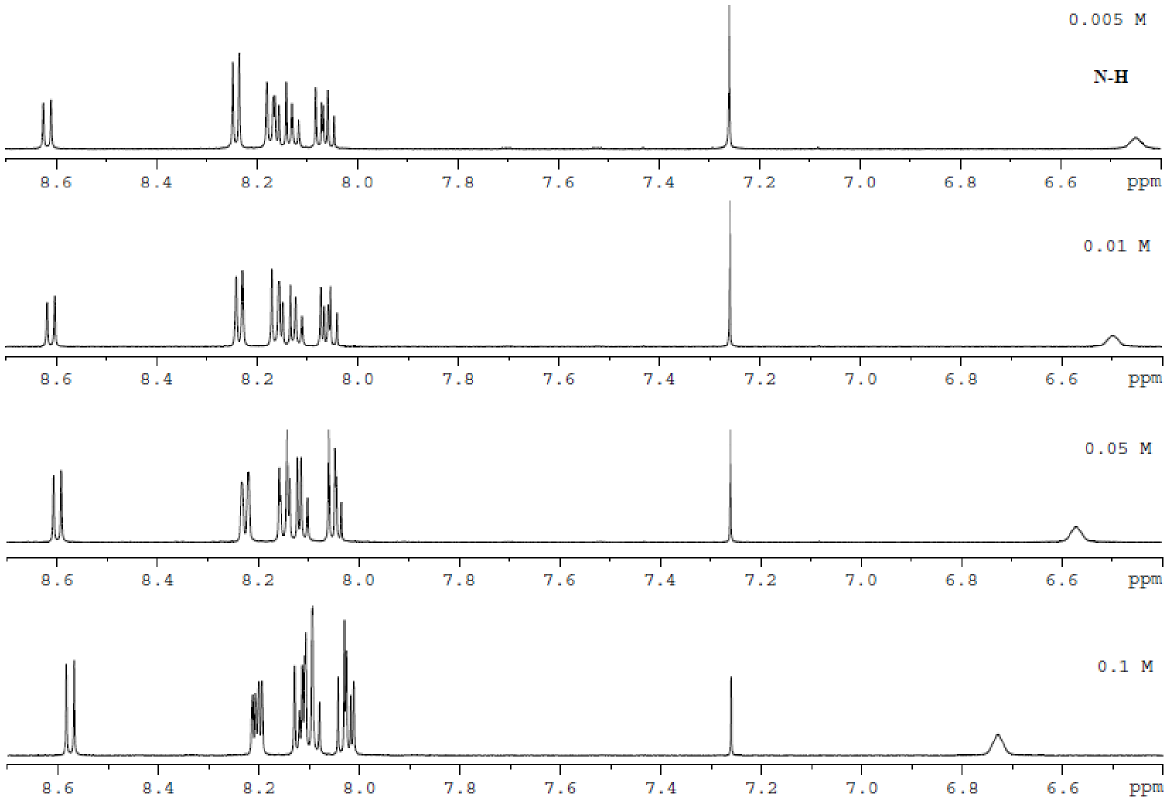

4. Photophysical study

Electronic absorption spectra were run on a Perkin Elmer Lambda 45 UV–vis spectrometer. Corrected emission spectra were obtained on a Fluorolog FL3-221 spectrofluorometer from Horiba Jobin-Yvon, including an integration sphere accessory which allows recording excitation and emission spectra and determining absolute quantum yield values in the powder state.

Fluorescence decay curves were obtained by the time-correlated single-photon counting (TCSPC) method with femtosecond laser excitation using a Spectra-Physics set-up composed of a Titanium Sapphire laser (Tsunami, Spectra-Physics) pumped by a doubled YAG laser (Millennia, Spectra-Physics), itself pumped by two laser diode arrays. Light pulses at 720 nm were selected by optoacoustic crystals at a repetition rate of 4 MHz, and then doubled to 360 nm by non-linear crystals. Fluorescence photons were detected through a monochromator by

means of a Hamamatsu MCP R3809U photomultiplier connected to a constant-fraction discriminator. The time-to-amplitude converter was purchased from Tennelec. The instrumental response function was recorded before each decay measurement. The fluorescence data were analysed using the Globals software package developed at the Laboratory for Fluorescence Dynamics at the University of Illinois at Urbana-Champaign, which includes deconvolution analysis and the global non-linear least-squares minimization method.

References

1. Psurski, M.; Błażewska, K.; Gajda, A.; Gajda, T.; Wietrzyk, J.; Oleksyszyn, J. *Bioorg. Med. Chem. Lett.* **2011**, *21*, 4572-4576. doi:10.1016/j.bmcl.2011.05.113
2. Sikora, D.; Gajda, T. *Phosphorus, Sulfur Silicon Relat. Elem.* **2000**, *157*, 201-210. doi: 10.1080/10426500008040523
3. Wrona-Piotrowicz, A.; Zakrzewski, J.; Metivier, R.; Brosseau, A.; Makal, A.; Wozniak, K. *RSC Adv.* **2014,** *4*, 56003-56012. doi:10.1039/C4RA07045C
